# Digital Eye Straining: Exploring Its Prevalence, Associated Factors, and Effects on the Quality of Life

**DOI:** 10.7759/cureus.59442

**Published:** 2024-05-01

**Authors:** Mohamed W Bin Maneea, Halah O Alamawi, Abdulaziz Almuqbil, Jana K Abukhlaled, Ghadah Alsuwailem, Jehad Alabdulminaim, Abdulrahman Mohammed M Aladawi, Asmaa Y Alshangiti

**Affiliations:** 1 Ophthalmology, Security Forces Hospital, Riyadh, SAU; 2 College of Medicine, King Abdullah bin Abdulaziz University Hospital, Riyadh, SAU; 3 College of Medicine, Imam Mohammad Ibn Saud Islamic University (IMSIU), Riyadh, SAU; 4 College of Medicine, Princess Nourah Bint Abdul Rahman University, Riyadh, SAU; 5 College of Medicine, King Saud University, Riyadh, SAU; 6 Medicine, Majmaah University, Majmaah, SAU; 7 College of Medicine, King Saud bin Abdulaziz University for Health Sciences, Riyadh, SAU

**Keywords:** eye strain factors, digital and eye, digital eye strain symptoms, eye strain, digital eye strain

## Abstract

Background: Digital eye strain (DES) has become a pervasive issue in contemporary society due to increased reliance on electronic devices. This study aims to comprehensively explore the symptoms, severity, and associated factors of DES, considering demographic, behavioral, and health-related variables.

Methodology: A cross-sectional study was conducted among participants with diverse demographic backgrounds. A structured questionnaire collected data on participant characteristics, electronic device usage patterns, symptoms of DES, and its impact on various aspects of quality of life. Statistical analyses, including chi-square tests, were employed to assess associations and significance.

Results: The majority of participants reported symptoms of DES, with eye dryness, headache, and eye redness being the most prevalent. Symptom severity varied, with age, daily device usage, adherence to the 20-20-20 rule, and studying with electronic devices demonstrating statistically significant associations. Participants diagnosed with eye diseases exhibited higher symptom severity. While disagreement was common regarding DES increasing stress, a substantial proportion acknowledged its impact on productivity and attention.

Conclusion: The current study showed that there is a significant correlation between the incidence of digital eye straining and longer screen exposure time. The findings underscore the importance of age, behavior, and ocular health in understanding and addressing DES. The results contribute to the broader discourse on digital eye health and emphasize the need for targeted interventions to alleviate the impact of DES on daily life.

## Introduction

A group of eye- and vision-related issues caused by extended use of a computer, tablet, e-reader, or mobile phone is referred to as computer vision syndrome, also known as digital eye strain (DES) [1,2]. When observing digital screens for prolonged durations, many people endure eye irritation and visual issues. With more time spent on digital screens, pain levels seem to increase [3,4].

Due to its special features and demanding visual requirements, using a digital screen or computer can result in vision-related complaints [1]. The intensity of computer vision syndrome (CVS) or what is also known as DES symptoms might be exacerbated by untreated visual issues. Possible exacerbating factors include computer screen's lettering, when the contrast is lower, and challenging view due to glare and reflections [1]. This work requires different viewing distances and angles than are often utilized for other reading or writing tasks. Uncorrected or inadequately fixed vision issues can significantly contribute to CVS. This will mostly lead to several symptoms related to vision and probably musculoskeletal issues [1]. These symptoms include headaches, impaired vision, and dry eyes. Many risk factors may contribute to these symptoms, including bad lighting, uncorrected refractive problems, glare on the digital screen, and an insufficient viewing distance. This syndrome can be caused by one or more of several factors. DES symptoms are usually reduced by getting regular eye care and changing how the screen is viewed. For example, customized spectacles may be provided to address the unique visual demands of computer viewing [1].

In another study conducted to estimate the prevalence of CVS among university medical students in Riyadh, Saudi Arabia, 94.0% reported at least one symptom of CVS, with 67% reporting more than three symptoms. CVS is common among medical students during COVID-19. This demands a greater understanding of CVS and its preventive actions [5]. Therefore, in this study, we aim to assess CVS prevalence, awareness, risk factors, and effect on quality of life among Riyadh City university students in Saudi Arabia.

## Materials and methods

The study was conducted in Saudi Arabia, spanning from September 2023 to October 2023, with a focus on Riyadh City universities. The target population included all students at the university, and participant information was kept confidential in accordance with ethical approval.

All students enrolled at Riyadh City universities were eligible for participation without restrictions based on age or other specifications. Excluded from the study were individuals who were not registered as students at Riyadh City universities.

An analytical retrospective cross-sectional approach was employed, utilizing a questionnaire-based survey. Data collection took place through an online survey distributed via university email and various social media platforms in Saudi Arabia. The survey link facilitated the retrieval of the required information.

The study determined a sample size of 253 to achieve a 96% confidence level with a 4% margin of error. An additional 20% was added to the sample size to ensure an adequate response rate, resulting in a final sample of 209 Riyadh University students. A convenient non-probability sampling method was employed, including individuals meeting the predefined inclusion and exclusion criteria. The research approval was obtained from King Abdulaziz City for Science and Technology (KACST) through Princess Nourah University (HAP-01-R-059).

The data were managed and analyzed using SPSS version 20 (IBM Corp., Armonk, NY). Descriptive statistics were applied to present categorical data, including percentages and frequencies, such as gender distribution. The relative risk was calculated to assess risks, along with the determination of confidence intervals. Logistic regression was utilized to evaluate potential risk factors. A significance level of 0.05 was set, considering the test as significant if the p-value fell below this threshold.

## Results

Table 1 outlines the demographic characteristics of the study participants. The majority of respondents were female (81.8%), with males accounting for 18.2%. Regarding age distribution, 76.6% fell within the 18-25 age range, while 21.1% were aged 26-35, and 2.4% were in the 36-45 age group. The distribution of participants across different colleges varied, with medical colleges having the highest representation at 35.4%. The majority of respondents held a bachelor’s degree (90.0%), followed by master’s degree holders (7.7%). In terms of daily sleeping duration, 50.2% reported sleeping for five to six hours, while 39.7% slept for six hours or more daily. Notably, 10.0% of the participants reported sleeping for less than four hours. A significant portion (51.2%) reported never using the 20-20-20 rule for screen time, while 50.2% adhered to a five-to-six-hour daily screen time. A majority of participants (89.5%) did not smoke. Concerning eye health, the majority reported never being diagnosed with any eye disease (31.6%). Refractive eye disorders were prevalent (37.3%), followed by eye dryness (30.1%) (Table 1).

**Table 1:** Demographic factors of the participants N: Frequency of the participants. N%: Corresponding percentages.

Variables		N (N = 209)	N %
Gender	Male	38	18.2%
	Female	171	81.8%
Age (years)	18-25	160	76.6%
	26-35	44	21.1%
	36-45	5	2.4%
College	Medical colleges	76	35.4%
	Computer science	41	19.6%
	Early childhood college	1	0.5%
	Law college	6	2.9%
	Faculty of Education	19	9.1%
	College of languages	8	3.8%
	Science	9	4.3%
	College of Business Administration	13	6.2%
	Engineering specializations	11	5.3%
	Arts and culture	9	4.3%
	Graphic science	7	3.3%
	College of Sports and Physical Activity	6	2.9%
	College of Literature	3	2.4%
Educational level	Diploma	5	2.4%
	Bachelor's	188	90.0%
	Master	16	7.7%
Daily sleeping duration	<4 hours	21	10.0%
	5-6 hours	105	50.2%
	6 hours or more	83	39.7%
Do you use the 20-20-20 rule (for every 20 minutes a person looks at the screen, they have to look at something 20 feet away for 20 seconds)?	Never	107	51.2%
	Rarely	30	14.4%
	Sometime	55	26.3%
	Usually	14	6.7%
	Always	3	1.4%
Do you use lubricating eye drops?	Never	49	23.4%
	Rarely	41	19.6%
	Sometime	69	33.0%
	Usually	35	16.7%
	Always	15	7.2%
Smoking	No	187	89.5%
	Yes	22	10.5%
Diagnosis with eye condition	Never diagnosed with any eye disease	66	31.6%
	Refractive eye disorders (such as nearsightedness and farsightedness)	78	37.3%
	Eye dryness	63	30.1%
	squint	18	8.6%
	Conjunctivitis	13	6.2%

Electronic device usage patterns are detailed in Table 2. The most common number of daily devices used was two (42.1%), and a majority (64.1%) reported always studying with electronic devices. Entertainment (54.1%) and reading (59.8%) were the most common uses of electronic devices. The majority (41.6%) reported using devices for six to eight hours per day. Regarding caffeinated beverage consumption, 39.2% reported consuming two cups daily, while 22.0% reported drinking one cup daily (Table 2).

**Table 2:** Electronic devices pattern of use N: Number of counts (or frequency). N%: Corresponding percentages to the count.

Variables	Pattern	Count (N)	Column (N %)
What is the total number of devices you use daily (including phones, tablets, laptops, computers, etc.)?	One	11	5.3%
	Two	88	42.1%
	Three	85	40.7%
	Four	16	7.7%
	Five	5	2.4%
	6 or more	4	1.9%
Do you study using electronic devices (iPad, tablet, Kindle, laptop, etc.)?	Never	2	1.0%
	Rarely	7	3.3%
	Sometimes	44	21.1%
	Usually	22	10.5%
	Always	134	64.1%
Uses of electronic devices	Searching	108	51.7%
	Reading	125	59.8%
	Data analysis	22	10.5%
	Technology-related work	47	22.5%
	Programming	40	19.1%
	Writing	104	49.8%
	Education	7	3.3%
	Data entry	41	19.6%
	Entertainment	113	54.1%
How many hours do you use your devices per day (total)?	1-3 hour	9	4.3%
	3-6 hours	43	20.6%
	6-8 hours	87	41.6%
	More than 8 hours	70	33.5%
How many cups of caffeinated beverages (tea, coffee, energy drinks, soft drinks) do you drink daily?	Never	28	13.4%
	One	46	22.0%
	Two	82	39.2%
	Three	35	16.7%
	Four or higher	18	8.6%

Table 3 further characterized digital eye straining based on reported symptoms. The majority of participants (67.0%) reported experiencing one to five symptoms, while 30.1% reported six to 10 symptoms. Only a small percentage (2.4%) reported no symptoms, and 0.5% reported more than 11 symptoms. Concerning frequency, most participants reported experiencing symptoms at varying intervals, with 40.2% reporting one to two times weekly and 17.2% reporting three to four times weekly (Table 3).

**Table 3:** Characteristics of digital eye straining N indicates the count or frequency of the variable. N% indicates the representing percentage of the count (N).

Variables		Count (N)	Column (N%)
Symptoms	No symptoms	5	2.4%
	1-5 symptoms	140	67.0%
	6-10 symptoms	63	30.1%
	>11 symptoms	1	0.5%
How frequent are the above symptoms?	Never having symptoms	5	2.4%
	Rarely (Less than 3 times monthly)	62	29.7%
	1-2 times weekly	84	40.2%
	3-4 times weekly	36	17.2%
	5-6 times weekly	9	4.3%
	Daily	13	6.2%
Preventive	Rest your eyes from time to time	91	43.5%
	Reduce screen brightness	157	75.1%
	Adjust your devices to a comfortable level	76	36.4%
	Put some distance between you and the device	61	29.2%

**Figure 1:**
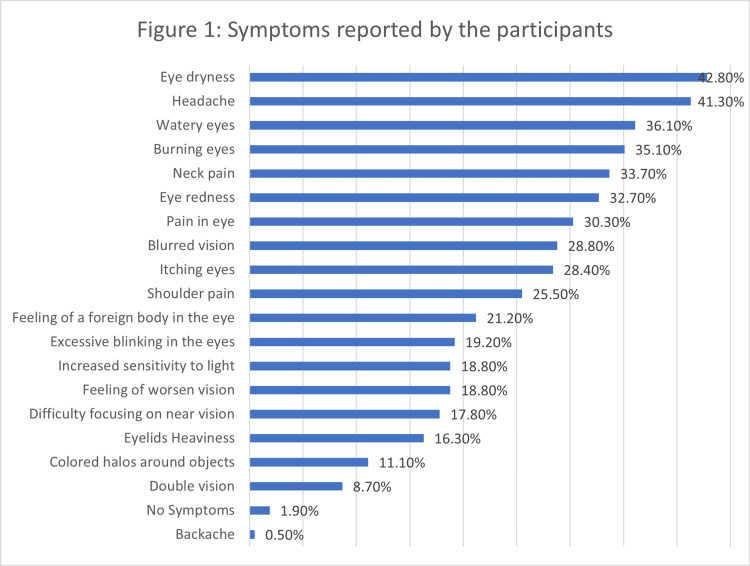
Symptoms reported by the participants

**Figure 2:**
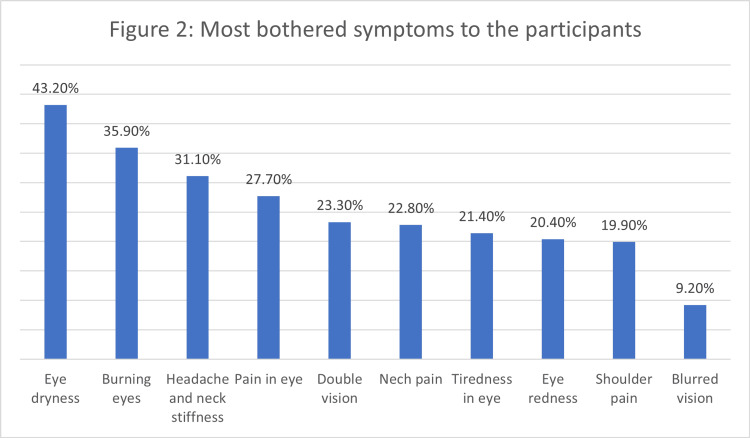
Most bothered symptoms to the participants

Figures 1, 2 highlight the symptoms that participants found most bothersome. Eye dryness (43.2%), burning eyes (35.9%), and head and neck stiffness (31.1 %) as well as pain in the eye (27.7%) were the top three most bothersome symptoms.

Table 4 delves into the impact of digital eye straining on the quality of life. A significant proportion (74.2%) disagreed that DES increased stress levels in their daily lives, while 25.8% agreed. Regarding productivity, 46.9% agreed that the symptoms affected their ability to complete tasks. Additionally, 57.4% agreed that DES reduced their level of attention or concentration. Notably, 51.7% of participants who suffered from migraines or chronic headaches reported that these conditions worsened with prolonged device use. Daytime sleepiness (56.3%) and difficult sleep entry (58.3%) were the most prevalent sleep-related issues reported.

**Table 4:** Impact of digital eye straining on the quality of life N: Frequency of affected participants. N%: Corresponding N in percentage form.

Impact on quality-of-life variables		N (total = 209)	N% (in percentage)
Digital eye strain increases the stress level in my daily life.	No	155	74.2%
	Yes	54	25.8%
The symptoms of digital eye strain affect my productivity and my ability to complete tasks.	Disagree	30	14.4%
	Neutral	81	38.8%
	Agree	98	46.9%
Digital eye strain increases the stress level in my daily life.	Disagree	48	23.0%
	Neutral	63	30.1%
	Agree	98	46.9%
Digital eye strain reduces my level of attention or concentration.	Disagree	30	14.4%
	Neutral	59	28.2%
	Agree	120	57.4%
Symptoms of digital eye strain greatly affect my driving.	Don’t have a car	55	26.3%
	Disagree	36	17.2%
	Neutral	53	25.4%
	Agree	65	31.1%
I feel that eye redness caused by digital stress is socially embarrassing.	Disagree	88	42.1%
	Neutral	64	30.6%
	Agree	57	27.3%
If you suffer from migraines or chronic headaches, do you get worse by using devices for long periods?	Disagree	29	13.9%
	Neutral	72	34.4%
	Agree	108	51.7%
The symptoms of digital eye strain affect the quality of my sleep.	Disagree	64	30.6%
	Neutral	49	23.4%
	Agree	96	45.9%

The relationship between the severity of digital eye straining and various demographic factors was explored. Analysis revealed that there were no statistically significant differences in the number of reported symptoms based on gender (p = 0.333), educational level (p = 0.398), and total number of devices used daily (p = 0.298). However, age showed a statistically significant association (p = 0.002), with the 26-35 age group reporting higher severity. Significant associations were observed between the severity of digital eye straining and the number of hours spent using devices daily (p = 0.009). Participants using devices for more than eight hours a day reported higher symptom severity. Additionally, the use of the 20-20-20 rule showed a statistically significant association (p = 0.036), with those who never used the rule reporting more severe symptoms. A statistically significant association was found between studying using electronic devices and symptom severity (p = 0.011), with those who always studied using electronic devices reporting higher severity. Participants diagnosed with any eye disease reported a statistically significant association with symptom severity (p = 0.009) (Table 5).

**Table 5:** The relationship between the severity of digital eye straining and demographic factors N: Frequency of participants. %: Percent of participants based on row count. * Significant at p-value lower than 0.05.

Variables		Number of symptoms reported by the participants								
		No symptoms		1-5 symptoms		6-10 symptoms		>11 symptoms		P-value
		N	Row N %	N	Row N %	N	Row N %	N	Row N %	
Gender	Male	2	5.3%	28	73.7%	8	21.1%	0	0.0%	0.333
	Female	3	1.8%	112	65.5%	55	32.2%	1	0.6%	
Age (years)	18-25	5	3.1%	94	58.8%	60	37.5%	1	0.6%	0.002*
	26-35	0	0.0%	41	93.2%	3	6.8%	0	0.0%	
	36-45	0	0.0%	5	100.0%	0	0.0%	0	0.0%	
Educational level	Diploma	0	0.0%	4	80.0%	1	20.0%	0	0.0%	0.398
	Bachelor's	5	2.7%	121	64.4%	61	32.4%	1	0.5%	
	Master	0	0.0%	15	93.8%	1	6.3%	0	0.0%	
What is the total number of devices you use daily (including phones, tablets, laptops, computers, etc.)?	One	1	9.1%	8	72.7%	2	18.2%	0	0.0%	0.298
	Two	2	2.3%	60	68.2%	26	29.5%	0	0.0%	
	Three	2	2.4%	55	64.7%	28	32.9%	0	0.0%	
	Four	0	0.0%	12	75.0%	3	18.8%	1	6.3%	
	Five	0	0.0%	3	60.0%	2	40.0%	0	0.0%	
	6 or more	0	0.0%	2	50.0%	2	50.0%	0	0.0%	
How many hours do you use your devices per day (total)?	1-3 hours	1	11.1%	6	66.7%	2	22.2%	0	0.0%	0.009*
	3-6 hours	0	0.0%	39	90.7%	4	9.3%	0	0.0%	
	6-8 hours	2	2.3%	58	66.7%	26	29.9%	1	1.1%	
	More than 8 hours	2	2.9%	37	52.9%	31	44.3%	0	0.0%	
Do you use the 20-20-20 rule (for every 20 minutes a person looks at the screen, they have to look at something 20 feet away for 20 seconds)?	Never	5	4.7%	60	56.1%	41	38.3%	1	0.9%	0.036*
	Rarely	0	0.0%	18	60.0%	12	40.0%	0	0.0%	
	Sometimes	0	0.0%	46	83.6%	9	16.4%	0	0.0%	
	Usually	0	0.0%	13	92.9%	1	7.1%	0	0.0%	
	Always	0	0.0%	3	100.0%	0	0.0%	0	0.0%	
Do you study using electronic devices (iPad, tablet, Kindle, laptop, etc.)?	Never	0	0.0%	2	100.0%	0	0.0%	0	0.0%	0.011*
	Rarely	0	0.0%	7	100.0%	0	0.0%	0	0.0%	
	Sometimes	0	0.0%	40	90.9%	4	9.1%	0	0.0%	
	Usually	0	0.0%	17	77.3%	5	22.7%	0	0.0%	
	Always	5	3.7%	74	55.2%	54	40.3%	1	0.7%	
Being diagnosed with any eye disease	Yes	0	0.0%	98	68.5%	44	30.8%	1	0.7%	0.009*
	No	5	7.6%	42	63.6%	19	28.8%	0	0.0%	

The association between the severity of digital eye straining and its impact on various aspects of quality of life was investigated. A significant association was found between symptom severity and increased stress levels in daily life (p = 0.000). Participants reporting symptoms were more likely to experience elevated stress. The impact on productivity and the ability to complete tasks was also significantly associated with symptom severity (p = 0.022), with those reporting more symptoms being more affected. The association between symptom severity and reduced attention or concentration (p = 0.259), the impact on driving (p = 0.142), social embarrassment due to eye redness (p = 0.236), worsening migraines or chronic headaches (p = 0.122), and the effect on sleep quality (p = 0.015) was not statistically significant (Table 6).

**Table 6:** The relationship between the severity of digital eye straining and the quality of life N: Frequency of participants. %: Percent of participants based on row count. * Significant at p-value lower than 0.05.

Variables		Number of symptoms reported by the participants								
		No symptoms		1-5 symptoms		6-10 symptoms		>11 symptoms		P-value
		N	Column N %	N	Column N %	N	Column N %	N	Column N %	
Digital eye strain increases the stress level in my daily life.	No	4	80.0%	119	85.0%	32	50.8%	0	0.0%	0.000*
	Yes	1	20.0%	21	15.0%	31	49.2%	1	100.0%	
The symptoms of digital eye strain affect my productivity and ability to complete tasks.	Disagree	1	20.0%	19	13.6%	10	15.9%	0	0.0%	0.022*
	Neutral	2	40.0%	66	47.1%	13	20.6%	0	0.0%	
	Agree	2	40.0%	55	39.3%	40	63.5%	1	100.0%	
Digital eye strain reduces my level of attention or concentration.	Disagree	2	40.0%	22	15.7%	6	9.5%	0	0.0%	0.259
	Neutral	1	20.0%	44	31.4%	14	22.2%	0	0.0%	
	Agree	2	40.0%	74	52.9%	43	68.3%	1	100.0%	
Symptoms of digital eye strain greatly affect my driving.	Not having a car	2	40.0%	29	20.7%	24	38.1%	0	0.0%	0.142
	Disagree	1	20.0%	30	21.4%	5	7.9%	0	0.0%	
	Neutral	1	20.0%	39	27.9%	13	20.6%	0	0.0%	
	Agree	1	20.0%	42	30.0%	21	33.3%	1	100.0%	
I feel that eye redness caused by digital stress is socially embarrassing.	Disagree	2	40.0%	53	37.9%	33	52.4%	0	0.0%	0.236
	Neutral	2	40.0%	49	35.0%	13	20.6%	0	0.0%	
	Agree	1	20.0%	38	27.1%	17	27.0%	1	100.0%	
If you suffer from migraines or chronic headaches, do you get worse by using devices for long periods?	Disagree	1	20.0%	25	17.9%	3	4.8%	0	0.0%	0.122
	Neutral	3	60.0%	48	34.3%	21	33.3%	0	0.0%	
	Agree	1	20.0%	67	47.9%	39	61.9%	1	100.0%	
The symptoms of digital eye strain affect the quality of my sleep	Disagree	2	40.0%	46	32.9%	16	25.4%	0	0.0%	0.015*
	Neutral	3	60.0%	38	27.1%	8	12.7%	0	0.0%	
	Agree	0	0.0%	56	40.0%	39	61.9%	1	100.0%	

## Discussion

The study delves into the realm of DES, starting with an exploration of the symptoms reported by the participants. Among the myriad symptoms, eye dryness, headache, and eye redness emerged as the most prevalent. These findings are consistent with established literature on DES symptoms [1,2,6,7]. These symptoms, along with others such as burning eyes, watery eyes, and itching eyes, collectively paint a vivid picture of the multifaceted impact of prolonged digital device usage on ocular health. Notably, participants also reported non-ocular symptoms like neck pain and increased sensitivity to light, emphasizing the systemic nature of the effects associated with DES [8,9].

Moving beyond the manifestation of symptoms, the study meticulously evaluates the severity of DES experienced by participants. The majority reported experiencing one to five symptoms, with only a small percentage indicating no symptoms. This spectrum of severity extends to those reporting six to 10 symptoms, while an even smaller group reported more than 11 symptoms. Understanding the distribution of symptom severity is essential for tailoring interventions and preventive strategies.

The study then navigates through the myriad factors that either contribute to or are affected by the severity of DES. Gender, educational level, and the number of daily devices used did not demonstrate statistically significant associations with symptom severity. This suggests that DES is a widespread issue affecting individuals across different educational backgrounds and genders. The lack of a significant association based on educational level contrasts with some studies linking higher education to increased screen time and digital device use [10,11]. In addition, some studies showed that female students were more affected by digital eye straining [6,12-14]. However, age emerged as a notable factor, with the 26-35 age group reporting higher severity compared to the 18-25 age group. This finding is in alignment with some studies that suggest older individuals may experience more symptoms due to age-related changes in eye physiology [2,8,15,16]. However, it aligns with the increasing trend of digital device usage among young adults, potentially contributing to higher symptom severity [17,18]. These findings challenge conventional notions that associate DES more closely with older age groups and suggest a nuanced interplay between age and technology-related behaviors.

Daily usage hours and adherence to the 20-20-20 rule exhibited statistically significant associations with symptom severity. Participants spending more than eight hours daily on devices reported higher symptom severity, underlining the role of prolonged exposure in the development of DES [2,19]. Similarly, those who never adhered to the 20-20-20 rule reported more severe symptoms, highlighting the potential protective nature of this rule [20,21].

The electronic device usage patterns reveal the ubiquity of technology in participants' lives. The most common number of daily devices used was two (42.1%), reflecting the multidevice usage trend in contemporary society. A significant majority (64.1%) reported always studying with electronic devices, emphasizing the integral role of technology in educational activities [22,23].

Entertainment (54.1%) and reading (59.8%) were identified as the primary uses of electronic devices. These findings align with broader societal trends where electronic devices serve not only as tools for work or study but also as sources of leisure and information [24].

The study also illuminates the association between studying using electronic devices and symptom severity. Participants who consistently studied with electronic devices reported higher severity, emphasizing the importance of considering educational practices in understanding and mitigating DES [6].

Further probing into the participants' health revealed intriguing associations. Individuals diagnosed with any eye disease exhibited a statistically significant link with symptom severity. This underscores the intricate relationship between pre-existing ocular conditions and the susceptibility to DES symptoms [19,25]. The association between migraines or chronic headaches and worsening symptoms with prolonged device use underscores the potential link between DES and broader health issues. Understanding these connections is crucial for comprehensive health management.

Intriguingly, the impact of DES on various aspects of quality of life was explored. While a significant proportion disagreed that DES increased stress levels, almost half agreed that the symptoms affected productivity and the ability to complete tasks. This suggests a complex interplay between the subjective experience of stress and the tangible effects on daily efficiency. The association between symptom severity and aspects like increased stress levels, reduced attention, and concentration was not statistically significant. This might indicate that individual differences, coping mechanisms, or external factors play a role in how DES manifests and impacts daily life [8,26].

Several limitations should be acknowledged. The study relied on self-reported data, introducing the possibility of recall bias. Objective measures, such as eye examinations, could provide more accurate information on eye health. Additionally, the cross-sectional design limits the establishment of causal relationships. Longitudinal studies tracking participants over time would offer insights into the progression of DES and its impact. Future research could delve into the effectiveness of specific interventions and preventive measures in mitigating DES symptoms. Exploring the role of factors like device ergonomics, blue light exposure, and individual visual habits could contribute to targeted interventions.

## Conclusions

In conclusion, this study provides a comprehensive overview of the prevalence, patterns, and impact of DES among a diverse group of participants. The findings underscore the multifaceted nature of DES, influenced by demographics, usage patterns, and health factors. Addressing DES requires a holistic approach encompassing awareness, preventive measures, and consideration of individual health contexts. This study contributes valuable insights into the evolving field of digital eye health and lays the foundation for further research and interventions.

## Appendices

**Table 7:** Digital eye straining questionnaire CVS: Computer vision syndrome.

(A) Demographics
Q1. Are you a university student in Riyadh?
Yes (will move to the questions)
No (The form will be submitted automatically)
Q2. Basic information
Age
18–25 years
26–35 years
36–45 years
Gender
Female
Male
What college major are you studying in?
Health-related specialties
Science
Computer sciences
Engineering
Arts and culture
Business administration
Social work
Education
Languages
Community college
Level of study
Diploma
Bachelor’s degree
Master’s degree
Doctorate
(B) Prevalence (Insert CVS Q questionnaire here)
a. Frequency
b. Intensity burning
Itching
A feeling of a foreign body
Tearing
Excessive blinking
Eye redness
Eye pain
Heavy eyelids
Dryness
Blurred vision
Double vision
Difficulty focusing for near vision
Increased sensitivity to light
Colored halos around objects
Feeling that eyesight is worsening
Headache
(C) Associated factors
How many devices do you use in total per day (including phones, tablets, laptops, and computers)?
One
Two
Three
Four
Five
More than 6
Do you study using electronic devices (iPads, tablets, kindle, laptops, etc.)?
Always
Usually
Sometimes
Rarely
Never
Are you diagnosed with any of the following eye diseases?
Refractive errors
Dry eye disease
Amblyopia
Uveitis
Glaucoma
Cataract
Conjunctivitis
Nonapplicable
How many hours per day do you use your devices (total)?
1-3 hrs
3-6 hrs
6-8 hrs
More than eight hours
Do you practice any of the following?
Data analysis
Programming
Research
Writing
Tech-related tasks
Data entry
Reading on devices
When using devices, do you do one of the following acts?
Resting your eye from time to time
Lower your screen brightness
Putting a distance between you and the device
Setting your devices on a comfortable level
How many cups of caffeinated drinks (tea, coffee, energy, soft drinks) do you drink per day?
None
One
Two
Three
More than four
How many hours per day do you sleep?
Less than four hours
5-6 hrs
6-8 hrs or more
Do you use the 20-20-20 rule (For every 20 minutes a person looks at a screen, they should look at something 20 feet away for 20 seconds)?
Always
Usually
Sometimes
Rarely
Never
Do you use lubricating eye drops?
Always
Usually
Sometimes
Rarely
Never
Do you smoke, use Sheesha, or vape?
Yes
No
(D) Common presentation
When you use a device for a long time, what are the most frequent symptoms you suffer from (you can choose multiple answers)?
Eye ache
Tired eyes
Burning
Dryness
Redness
Tearing and irritation
Blurred vision
Double vision
Shoulder pain
Neck pain
Neck stiffness and headache
(E) Effects on quality of life
Which of the following digital eye straining symptoms is most bothersome for you?
Eye ache
Tired eyes
Burning
Dryness
Redness
Tearing and irritation
Blurred vision
Double vision
Shoulder pain
Neck pain
Neck stiffness and headache
Digital eye straining symptoms are affecting my productivity and my ability to complete tasks.
Agree
Neutral
Disagree
Digital eye straining increases my daily life stress level.
Agree
Neutral
Disagree
If you suffer from migraine or chronic headaches, is prolonged device exposure a trigger for your condition?
Agree
Neutral
Disagree
Digital eye straining decreases my level of attention or concentration.
Agree
Neutral
Disagree
Digital eye straining symptoms significantly affect the car driving experience.
Agree
Neutral
Disagree
When digital eye straining symptoms cause red eyes, it is socially embarrassing for me.
Agree
Neutral
Disagree
